# Bush medicine of the Mbabaram Aboriginal community in Far North Queensland demonstrates strong antioxidant and anti-inflammatory activities

**DOI:** 10.1186/s12906-025-05042-2

**Published:** 2025-10-24

**Authors:** Gerry Turpin, Karma Yeshi, Darren Crayn, Karen Guivarra, Valmai Turpin, Shane Motlap, Phurpa Wangchuk

**Affiliations:** 1 Australian Tropical Herbarium (ATH), James Cook University, Nguma Bada Campus, McGregor Road, Smithfield, QLD 4878 Australia; 2 Queensland Herbarium and Biodiversity Science, Department of the Environment, Tourism, Science and Innovation (DETSI), Brisbane Botanic Gardens, Mount Coot-tha, Mount Cooth-Tha Road, Toowong, QLD 4066 Australia; 3 College of Science and Engineering (CSE), James Cook University, McGregor Rd, Smithfield, Cairns, QLD 4878 Australia; 4 Australian Institute of Tropical Health and Medicine (AITHM), James Cook University, McGregor Rd, Smithfield, Cairns, QLD 4878 Australia; 5 ARC Centre of Excellence for Indigenous and Environmental Histories and Futures, James Cook University, Nguma-Bada Campus, McGregor Rd, Smithfield, Cairns, QLD 4878 Australia; 6 Australian National Herbarium, Commonwealth Industrial and Scientific Research Organisation (CSIRO), Clunies Ross Street, ACT, Canberra, 2601 Australia; 7 Mbabaram Aboriginal Corporation (MAC), Shop 3, 30 Mabel Street, Atherton, QLD 4883 Australia

**Keywords:** Medicinal plants, Mbabaram Aboriginal Community, Anti-oxidant, Anti-inflammatory, Phytochemicals, Queensland, Australia

## Abstract

**Introduction:**

The Mbabaram Aboriginal community lives in Atherton Tableland of Far North Queensland, Australia. While most of the knowledge had been lost due to colonial influences, this community still has remnants of traditional biocultural knowledge, which is critically endangered. They have been closely working with the Tropical Indigenous Ethnobotany Centre (Queensland Herbarium, James Cook University) in the areas of documenting traditional biocultural knowledge and biodiscovery projects. The current study investigated five medicinal plants used by the Mbabaram Aboriginal community for treating wounds, and inflammation-associated diseases.

**Methods:**

In this study, crude extracts of five medicinal plants from the Mbabaram community (*Breynia oblongifolia, Cajanus reticulatus, Dodonaea lanceolata, Exocarpos latifolius,* and *Coleus amoenus*) were assessed for their phytochemical contents. The antioxidant activity was determined using the 2,2-diphenyl-1-picrylhydrazyl (DPPH) radical scavenging assay. Furthermore, crude extracts were evaluated for their effect on cell viability and anti-inflammatory activities using the human peripheral blood mononuclear cells (PBMC) assay.

**Results:**

While some plants tested positive for flavonoids and saponins, *B. oblongifolia* and *C. amoenus* did not test positive for saponins. Only *C. reticulatus* and *E. latifolius* tested positive for alkaloids. The water extract of *C. amoenus* and the ethanol extract of *B. oblongifolia* exhibited the highest TPC with 99.88 ± 4.47 GAE/g extract and 128.36 ± 14.09 GAE/g extract, respectively. While the crude water extract of *E. latifolius* stems showed the best antioxidant activity with EC_50_ value of 0.024 μg/mL, the water extract of *B. oblongifolia* leaf showed the best anti-inflammatory activity by significantly reducing the levels of four pro-inflammatory cytokines, namely IL-1β, IL-6, IL-8, and TNF, which are known for instigating IBD pathogenesis.

**Conclusions:**

Of the five aqueous crude extracts studied here, *E. latifolius* stems showed the best antioxidant activity and *B. oblongifolia* leaf showed the best anti-inflammatory activity. This result validated the traditional uses of medicinal plants, which is used for treating inflammation-related conditions including wounds and sores. *B. oblongifolia* has potential to yield drug lead molecules for developing treatment for inflammation and sores/ulcers related diseases such as IBD.

## Introduction

Nature and human culture, while often considered as two separate domains in environmental stewardship, are significantly interlinked with similar evolutionary paths, mutual adaptation and co-evolution [9]. The Australian Aboriginal and Torres Strait Islander people have lived within the ecology of Australia for thousands of years, adapting to changing climates and environments while developing a highly complex and intimate biocultural knowledge of the lands, seas and skies. One of this biocultural knowledge is ethnomedical plant knowledge which is often called customary medicine (CM) in Australia, a term that acknowledges the integration of traditional and contemporary use of medicinal plants. CM, especially as practised by the Mbabaram community in the Atherton Tablelands of Far North Queensland (FNQ), is a relatively untapped source of wisdom in biodiscovery projects [20].

The Mbabaram (Bar-Barrum) peoples’ traditional lands extend to the top of the Great Dividing Range near the Walsh River Spring, bounded by the Walsh River near Dimbulah and includes the present towns of Watsonville, Irvinebank, Almaden and south to Mt Garnet. Mbabaram is one of the 250 Australian languages spoken by Aboriginal and Torres Strait Islander people [17] used for communication and describing flora and fauna, which is also the foundation of bush medicine knowledge. Both the language and biocultural knowledge is critically endangered. In our continuous efforts to document, preserve and add commercial value to Mbabaram biocultural knowledge, we interviewed Mbabaram elders and knowledgeable non-indigenous people and documented the associated species names, common names, and cultural uses on Mbabaram bush medicine plants. Additional knowledge on Mbabaram plant names and uses was also retrieved from explorer journals, botanical textbooks, and from museum artefacts.

This knowledge collected, retrieved and transcribed was entered into a Miromaa database (Miromaa Aboriginal [13]), which is a simple, password-protected software platform enabling the control by Aboriginal knowledge holders of language and cultural knowledge. Research protocols were established between the community and Traditional Indigenous Ethnobotany Centre (TIEC) to govern the maintenance, control, protection, and development of Mbabaram traditional knowledge. Development of these protocols was undertaken in the context of the Queensland Biodiscovery Act 2004 and the recently amended traditional knowledge guidelines and code of practices [16]. This has improved alignment of legislation with international standards such as the Nagoya Protocol on Access and Benefit Sharing [4], which requires: i) biodiscovery to be undertaken only with the prior informed consent of Indigenous communities who hold traditional knowledge about the resources, and ii) the benefits of biodiscovery be fairly and equitably shared.

These protective and supportive initiatives towards Indigenous knowledge and our documentation projects ignited the Mbabaram community’s interest and aspirations to think about preparing bush food and bush medicines for commercial benefits. In response to these interests and aspirations of the Watsonville Mbabaram clan group, the TIEC at James Cook University (JCU) led a joint collaborative medicinal plant project, which was funded by an National Health and Medical Research Council (NMHRC). Ideas grant in 2020. This project has steered many developments and has resulted in a number of significant outcomes that has benefited both JCU and Mbabaram Aboriginal community. One of the major outcomes was this study, in which we have botanically identified and collected five medicinal plants from the Watsonville region of Far North Queensland, and tested them for their phytochemical content, toxicity, antioxidant and anti-inflammatory activities using advanced techniques. These five plants were used for healing wounds, sores, and toothache and treating inflammatory-related conditions.

The three plants showed potent antioxidant and anti-inflammatory activities by suppressing the pro-inflammatory cytokines responsible for the pathogenesis of human inflammatory bowel disease (IBD). The IBD has no cure, and the existing treatments comes at huge socio-economic cost and the unwanted side effects. Therefore, our study has identified three potential anti-inflammatory plant candidates that can be employed for biodiscovery and drug development.

## Materials and methods

### Study site and medicinal plant collection

**Fig. 1:**
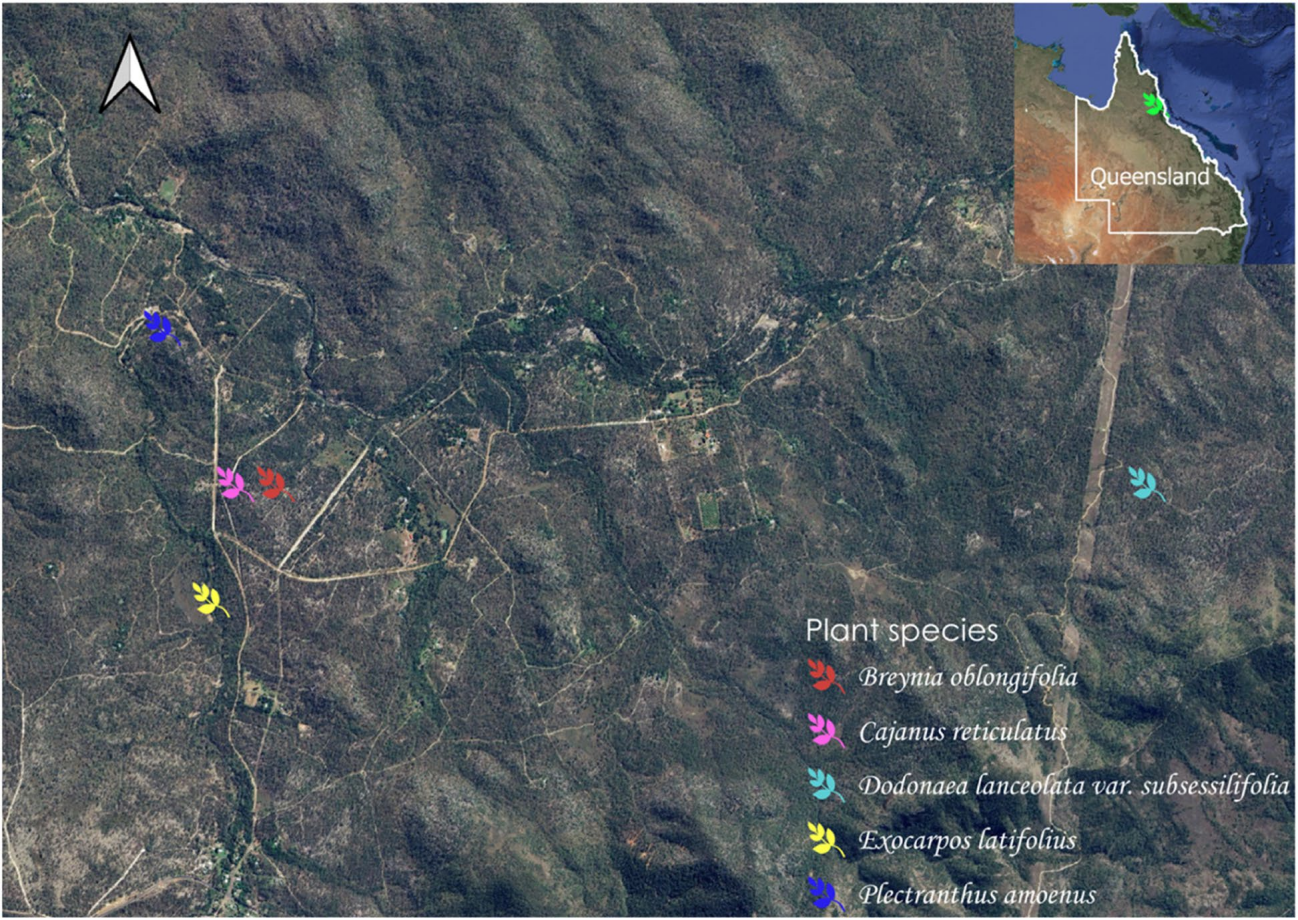
A map of Queensland, Australia, showing the collection sites of five medicinal plants. GPS coordinates for *Breynia oblongifolia* (latitude:17.3569; longitude:145.3150); *Cajanus reticulata* (latitude:17.3569; longitude:145.3130); *Dodonaea lanceolata var. subsessifolia* (latitude:17.3569; longitude:145.3580); *Exocarpos latifolius* (latitude:17.3626; longitude: 145.3118); *Coleus amoenus* (latitude:17.3492; longitude:145.3094); Plant coordinates were obtained while collecting the plant samples

The human ethics approval (H8072) and the plant collection permits (BIBC20200417-2) were obtained from the Department of Environment and Science, Queensland government. The leaves, stems, wood and roots of the five medicinal plant species traditionally used by Mbabaram Indigenous communities for healing/treating inflammation and inflammatory-related conditions were collected by a trained Aboriginal Ethnobotanist – Mr. Gerry Turpin (first author of this paper) from Watsonville region, Queensland, Australia (Fig. 1). The plants were identified by Mr. Gerry Turpin and Prof. Darren Crayn (Taxonomist) at the Australian Tropical Herbarium and the Queensland Herbarium, Brisbane. Herbarium specimen numbers were assigned to each plant species (Table 1), and samples were deposited at Australian Tropical Herbarium (ATH). Collected fresh plant materials were washed and dried.

**Table 1:** Mbabaram Aboriginal community uses of medicinal plants included for phytochemical and biological activity analysis in this study

Collection Number	Plant species and family	Common name	Parts used	Traditional uses	Location
MB6	*Breynia oblongifolia* (Müll.Arg.) Müll.Arg. (Phyllanthaceae)	Coffee Bush	Leaves	Sores	Bischoff Mill Rd, N of Watsonville, Qld
MB5	*Cajanus reticulatus* (Aiton) F.Muell. (Leguminosae)	Cajanus	Whole plant	Toothache	Bischoff Mill Rd, N of Watsonville, Qld
MB8	*Dodonaea lanceolata var. subsessilifolia* J.G.West (Sapindaceae)	Hop Bush	Leaves, stems, roots	Wounds	Creek W of Silver Valley Road, km W of Herberton, Qld
MB2	*Exocarpos latifolius* R.Br. (Santalaceae)	Broad-leaved Cherry	Leaves, stems, bark	Sores	Bischoff Mill Rd, N of Watsonville, Qld
MB3	*Coleus amoenus* P.I.Forst. (Lamiaceae)	Coleus	Leaves, stem	Wounds	Bischoff Mill Rd, N of Watsonville, Qld

### Preparation of crude plant extracts

Crude extracts were prepared using the protocol described by us previously [24]. Briefly, dried samples of the five medicinal plants (10 g each) were ground into a coarse powder using a nutriBullet grinder (NutriBullet, Australia). The coarse powder of each plant was macerated with ethanol (80%) three times for 20 min (~ one-hour total extraction time), and the fresh solvent was replaced at each 20 min interval. The extracts from three rounds of extraction were combined and filtered with a Whatman filter paper (Whatman Asia Pacific, San Centre, Singapore). Then, the ethanol extract was concentrated with the help of a rotary evaporator (Heidolph, Germany) and the aqueous extracts were frozen in − 80 °C freezer and subsequently freeze-dried (LABCONCO, John Morris Group).

### Determining major groups of phytochemicals

From the concentrated ethanol extract of each plant, a total volume of 16 mL of extract was used for the qualitative phytochemical screening tests following methods adapted from Wangchuk et al [21, 22], Iqbal et al. [8], Shah and Hossain [18], and Yeshi et al. [24]. Briefly, the crude extracts were analysed for major phytochemical classes, including alkaloids, flavonoids, tannins, terpenoids, glycosides, steroids, anthraquinones, and saponins.

The parent ethanol extract solution (16 mL) was divided into 8 parts (8 parts × 2 mL) and conducted the following eight tests:

1. Alkaloid test: Dragendorf’s reagent (1 mL) was added to the crude plant extract solution (2 mL). An orange precipitate confirmed the presence of alkaloid.
2. Flavonoid test: Hydrolysed with KOH and then added basic lead acetate (25%). A yellow precipitate indicated the presence of flavonoids class of phytochemicals.
3. Tannin test: To 2 mL crude extract solution, a few drops of 10% aqueous ferric chloride was added. The formation of a black precipitate or blue-green coloration confirmed the presence of tannin.
4. Terpenoid test: To the plant extract solutions (2 mL), chloroform (2 mL) was added, followed by a few drops of concentrated sulphuric acid. The formation of reddish-brown coloration at the interface confirmed the presence of terpenoid.
5. Cardiac glycoside test: Glacial acetic acid (2 mL) was added to the 2 mL plant crude extract solution, followed by a few drops of ferric chloride (10%) and then sulphuric acid (5%, 1 mL). A brown ring was formed at the interface of solvents confirming the presence of cardiac glycoside.
6. Anthraquinone glycoside test: Sulphuric acid (5%, 1 mL) and chloroform (2 mL) were added to a 2 mL extract solution followed by adding few drops of diluted ammonia to the lower layer of solution. Rose pink to red colour of the lower ammoniacal layer confirmed the presence of anthraquinone glycosides.
7. Steroid test: A chloroform (2 mL) was added to the crude extract solution (2 mL). A few drops of acetic anhydride were added, and the mixture was boiled in a water bath. The solution was cooled in the ice water and a concentrated sulphuric acid (1 mL) was then added to it. The formation of a brown ring at the junction/solvent interface and change of the upper layer into green confirmed the presence of steroid.
8. Saponin test: A distilled water (1 mL) was added to the crude extract solution (2 mL) and was shaken for few minutes. The presence of more than 1 cm foam/frothing that persisted after warming in the water bath (37 °C) confirmed the presence of saponin.

### Determination of Total Phenolic Content (TPC

The Folin-Ciocalteu (F–C) method was used to determine the total phenolic content (TPC) in crude extracts of the five medicinal plants following the slightly modified methods described by Molole et al. [14], and Yeshi et al. [24]. Briefly, 1 mg crude extracts were dissolved in 1 ml of 80%ethanol to prepare a solution. 100 μl of sample was aliquoted/dissolved in 500 μl of the Folin–Ciocalteu reagent. The aliquat was mixed with 2 mL of a freshly prepared F–C reagent (diluted with distilled water at a 1:10 ratio) in triplicates. After three but not more than eight minutes, 4 mL of sodium carbonate (7.5% w/v in distilled water) was added. Then, the reaction mixture was incubated at room temperature for 30 min. After 30 min incubation, absorbance was recorded at 760 nm. The gallic acid (purchased from Sigma Aldrich, Victoria, Australia) standard calibration curve (0–150 µg/mL concentrations in triplicates) was prepared to calculate the TPC following the same procedure. Finally, the TPC was expressed in terms of milligrams of gallic acid equivalent per gram of dry extract (mg GAE/g) from the equation below [19]:

$$\text{C}=\text{C}1 \times \frac{\textrm{V}}{\textrm{m}}$$

where ‘C’ = TPC in mg GAE/g extract, ‘C_1_’ = the concentration of gallic acid determined from the calibration curve in mg/mL, ‘V’ = the volume of the extract in mL, and ‘m’ = the weight of the dry plant extract in g.

### Determination of antioxidant activity of crude extracts

The capacity of the crude extracts to scavenge DPPH-free radicals was examined using the modified methods from Brand-Williams et al. [3], Yeshi et al. [24], and Molole et al. [14]. Briefly, various concentrations of 25, 100, 250, 500, 1000 μg/mL of crude extracts were prepared by dissolving them in the 80% aqueous ethanol. 100 μL of sample extract or 100 μL of the positive control (gallic acid, at the same concentrations) were mixed with 150 uL of DPPH (0.1 mM in an ethanol solution) and placed in a flat-bottom 96-well plate (Falcon®, Corning, NJ, USA). The sample aliquots were prepared in triplicates and mixed well with 0.1 mM methanolic DPPH solution 1:2 ratio. The reaction mixture was incubated in the dark at room temperature for 1 h, and following the incubation, their absorbance was measured at 517 nm using a microplate reader (SPECTROstar® Omega, BMG Labtech, Mornington, Australia). Methanol with DPPH was used as a negative control, and methanol as a blank. The percentage of scavenging activity was calculated from their absorbance readings using the formula:

$$\text{Percentage of DPPH free radical scavenged }(\%)=\frac{\textrm{Ac}-\textrm{As}}{\textrm{Ac}}\times 100{\%}$$

where A_c_ is the absorbance of the control and A_s_ is the absorbance of the sample/gallic acid.

To determine EC_50_ values (half maximal effective concentration), the dose–response DPPH-free radical scavenging activity was determined at various concentrations. Briefly, 100 μL of sample extract (25, 100, 250, 500, 1000 μg/mL) or 100 μL of the positive control (gallic acid, at the same concentrations) were mixed with 150 uL of an ethanol solution of DPPH (0.1 mM) and placed in a 96-well flat-bottom plate (Falcon®, Corning, NJ, USA). The dose–response curve was plotted with the obtained data, and EC_50_ values were determined by interpolating the standard curve (asymmetric sigmoidal, five-parameter logistic equation, 5PL), where ‘X’ is concentration using a GraphPad prism (v.10.0). The antioxidant activity was determined by.

### Determining anti-inflammatory activity of crude extracts

The *in-vitro* anti-inflammatory activity of the crude extracts were determined using a protocols described by us previously [24]. Briefly, the healthy human peripheral blood mononuclear cells (PBMCs) were supplied by the Australian Red Cross Lifeblood (two donors) under human ethics approval number H8523 (approved by the Human Research Ethics Committee, JCU). The PBMCs were cryopreserved in Foetal Bovine Serum (filtered heat-inactivated) (FBS—Corning #35–076-CV) containing 10% dimethyl sulfoxide (DMSO, Sigma-Aldrich). The PBMCs were separated using the Ficoll-Paque PLUS (GE Health) density gradient (STEMCELL technologies).

On the day of the experiment, PBMCs were thawed and washed with R-10 media (RPMI-1640 (Gibco), containing 10% heat-inactivated FBS, 100 U/mL Penicillin, and 100 μg/mL Streptomycin (Gibco). For the assay, 1 × 10^6^ cells in 100 μL of R-10 media were seeded per well in the 96-well U-bottom culture plates (Falcon®, Corning, NY, USA). For stimulated conditions, PBMCs were stimulated with lipopolysaccharide (LPS, 10 ng/mL) (Sigma-Aldrich) and incubated inside the incubator (PHCBI Corporation at 37 °C in a 5% CO_2_) for 2 h. After 2 h of LPS stimulation, PBMCs in respective treatment wells were treated in triplicates with the aqueous crude extracts of five medicinal plants (100 μg/mL in cell culture media with 0.5% DMSO). The R-10 media with DMSO was used as the negative control. Culture plates were incubated overnight at 37 °C in a 5% CO_2_ incubator. Following overnight incubation, plates were centrifuged (1500 rpm at 4 °C for 5 min), and the culture supernatants were collected for cytokine analysis. The remaining cells in the culture plates were stained and analysed further to analyse the effect of tested aqueous crude extracts on cell viability, as described below.

Culture supernatants were analysed (in triplicates for each treatment and control group) using a customised human 13-plex LEGENDplex™ multi-analyte flow assay kit (BioLegend®, USA) to quantify the cytokines released (pg/mL) by the LPS-stimulated PBMCs. This assay kit allows simultaneous detection of 13 human pro-inflammatory cytokines/chemokines, including interleukin (IL)−1β, interferon alpha (IFN-α), IFN-γ, tumor necrosis factor (TNF), monocyte chemoattractant protein-1 (MCP-1), IL-6, IL-8 (CXCL8), IL-10, IL-12p70, IL-17A, IL-18, IL-23, and IL-33 with the help of a flow cytometer (BD Biosciences). Flow cytometry data files were analysed in a cloud-based software platform from the BioLegend® website (San Diego, CA, USA). The concentrations of cytokines obtained in picograms per millilitres (pg/mL) were further normalised to the LPS-stimulated control (100%) and expressed as mean ± standard error (SEM). The LSRFortessa X20 flow cytometer (BD Biosciences) was used to acquire data for determining the anti-inflammatory activity of selected medicinal plants.

### Determining the effect of crude extracts on cell viability

Briefly, cells were stained with live/dead viability dye (LIVE/DEAD™ fixable near-IR Dead Cell Stain Kit for 633 or 635 nm excitation, cat. No. L2652701) as per the kit’s instruction. Stained cells were kept on ice in the dark for 30 min. After 30 min, stained cells were washed twice with Dulbecco’s phosphate-buffered saline (DPBS, Gibco) containing 2% FBS. Following the wash, cells were resuspended in a DPBS 2% FBS and ran through the flow cytometer (LSRFortessa X20, BD Biosciences). The data obtained from the flow cytometer (in FCS format) was further analysed using FlowJo (10.8.1 version software) to determine the percentage of live/Dead dye-positive cells. Culture supernatants of aqueous crude extracts that did not affect the cell viability were further analysed to quantify the pro-inflammatory cytokines.

### Statistical analysis

We performed the data analysis as per the protocols described by Yeshi et.al (2022). Data were further analysed by a one-way (ANOVA) in GraphPad Prism version 8.4.3 (San Diego, CA, USA), and the group means were compared using Dunnett’s Multiple Range Test. *p* < 0.05 was considered significant. Anti-inflammatory data were expressed as the mean ± SEM from two independent experiments.

## Results and discussion

### Ethnopharmacological uses of five medicinal plants

**Fig. 2:**
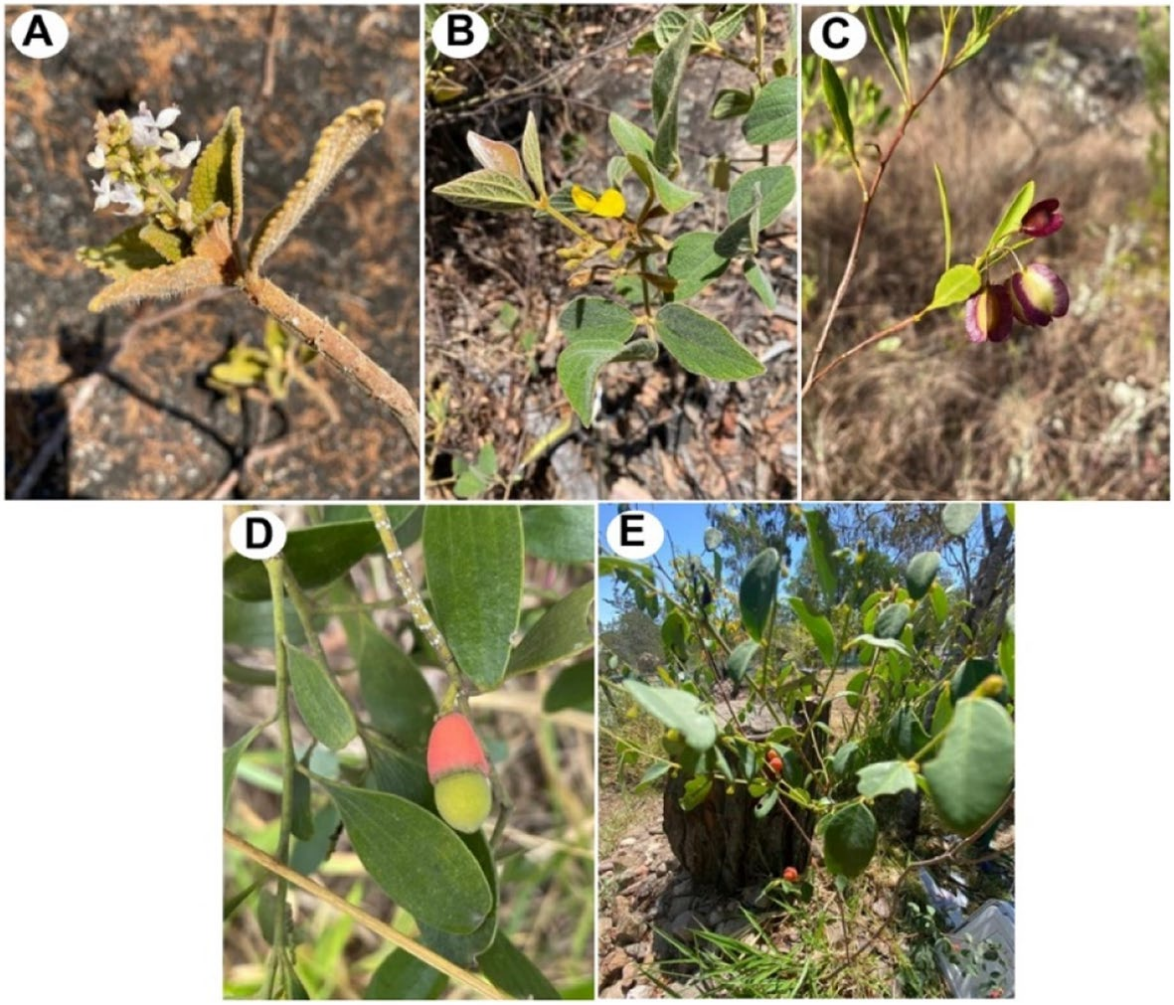
Aerial parts of five medicinal plants investigated in this study. (A) *Coleus amoenus,* (B) *Cajanus reticulatus,* (C) *Dodonaea lanceolata,* (D) *Exocapos latifolius,* and (E) *Breynia oblongifolia*. (Photo courtesy: G. Turpin)

The five medicinal plants or bush medicine used by the Mbabaram Aboriginal community for treating wounds, sores, and toothache were collected from places listed in Table 1 and their photos were presented in Fig. 2. We collected plant parts based on the ethnopharmacological uses of the plants by the community. For example, leaves, stems, and roots of *Dodonaea lanceolata var. subsessilifolia* J.G.West are used, leaves and stems of *Breynia oblongifolia* (Müll.Arg.) Müll.Arg. are used, and for *Coleus amoenus* P.I.Forst only leaves are used. Lukhoba et al. [11] indicated that 20 other species of *Coleus* are used for treating wounds and sores in other parts of the world, including India, Brazil, and Africa. *Breynia* species are also traditionally used for medicinal purposes in China, Philippines, and Indonesia [2, 7].

### Major groups of phytochemicals detected in the crude extracts of five medicinal plants

Ethanolic crude extracts from the five medicinal plants were analysed for major phytochemical classes, including alkaloids, flavonoids, tannins, terpenoids, glycosides, steroids, anthraquinones, and saponins. All tested positive for flavonoids, and all except *Breynia oblongifolia* and *Coleus amoenus* tested positive for saponins. Only *Cajanus reticulatus* and *Exocarpos latifolius* tested positive for alkaloids, and none tested positive for anthraquinones (Table 2). Of the five plants, *Dodonaea lanceolata var. subsessilifolia* showed positive results for the widest range of phytochemical classes (Table 2). Prior to this study, little was known of the phytochemistry of these five medicinal plant species. At the genus level, *Coleus* is known to contain mostly diterpenoids and phenolic compounds [1, 11]. More than 90 compounds have been isolated from the genus *Breynia,* and glycosides are the major constituents [2].

**Table 2:** The screening of crude extracts from five medicinal plants for the presence of major classes of phytochemicals

Plant species	Sample tested	Alka	Flavon	Tan	Terpen	Cardiac glycosid	Anthraq glycosid	Stero	Sapo
*Breynia oblongifolia*	Leaf	-	+	-	-	-	-	-	-
*Cajanus reticulatus*	Whole	+	+	-	+	-	+	+	+
*Dodonaea lanceolata var. subsessilifolia*	Leaf	-	+	+	+	+	-	-	+
	Stem	-	+	+	+	-	-	-	+
	Root	-	+	+	+	-	-	-	+
*Exocarpos latifolius*	Leaf	+	+	-	-	+	-	-	-
	Stem	+	-	-	-	-	+	-	-
	Bark	+	-	-	-	-	-	+	+
*Coleus amoenus*	Stem	-	+	-	-	-	-	+	-
	Root	-	-	-	-	+	-	+	-

Symbols: − indicates negative test for the phytochemical class tested; + indicates positive test for the phytochemical class tested. *Alka* alkaloids, *Flavon* flavonoids, *Tan* tannins, *Terpen* terpenoids, *glycosid* glycosides, *Anthraq* anthraquinone, *stero* steroids, *Sapo* saponins

### Total phenolics content (TPC) of five medicinal plant extracts

**Fig. 3:**
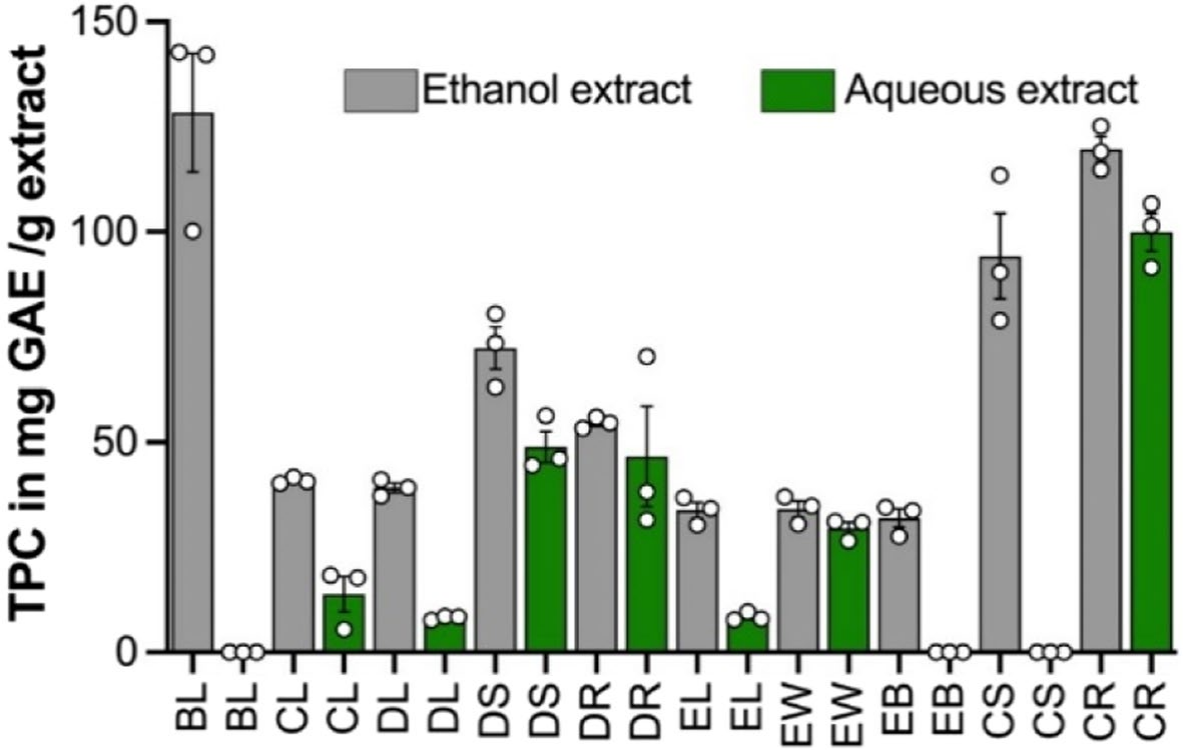
TPC of various parts of five medicinal plants extracted using ethanol (grey bar) and water (green bar). TPC is expressed in mg gallic acid equivalent (GAE)/g extract and shown as mean ± SEM (*n* = 3). BL-*Breynia oblongifolia var. subsessilifolia* leaf; CL-*Cajanus reticulatus* leaf; DL-*Dodonaea lanceolata var. subsessilifolia* leaf; DS-*Dodonaea lanceolata var. subsessilifolia* stem; EL-*Exocarpus latifolius* leaf; EW-*Exocarpos latifolius* wood; EB-*Exocarpus latifolius* bark; CS-*Coleus amoenus* stem; CR-*Coleus amoenus* root

The total phenolic content (TPC) of the crude extracts was determined by the Folin-Ciocalteu method and expressed in mg of gallic acid equivalent per gram of dried crude extract (GAE/g extract). The water and ethanol extracts of the leaves were prepared and for some plants, stems and roots were also prepared. Of the ethanol extracts, the highest TPC was found in *Breynia oblongifolia* leaf (128.36 ± 14.09 GAE/g extract), followed by *Coleus amoenus* root and stem with 119.67 ± 3.03 and 94.23 ± 10.16 GAE/g extract, respectively. Of the aqueous extracts, the *C. amoenus* root extract showed the highest TPC (99.88 ± 4.47 GAE/g extract), followed by *Dodonaea lanceolata var. subsessilifolia* stem extract, and *Exocarpos latifolius* root extract with 48.96 ± 3.70 and 46.66 ± 11.96 GAE/g extract, respectively (Fig. 3). Overall, *Coleus amoenus* had the highest TPC for the ethanol and aqueous extracts. After determining the qualitative and quantitative phytochemical contents, crude extracts were examined for their antioxidant activity using the DPPH-free radical scavenging assay.

### Antioxidant activities of five medicinal plant extracts

**Fig. 4:**
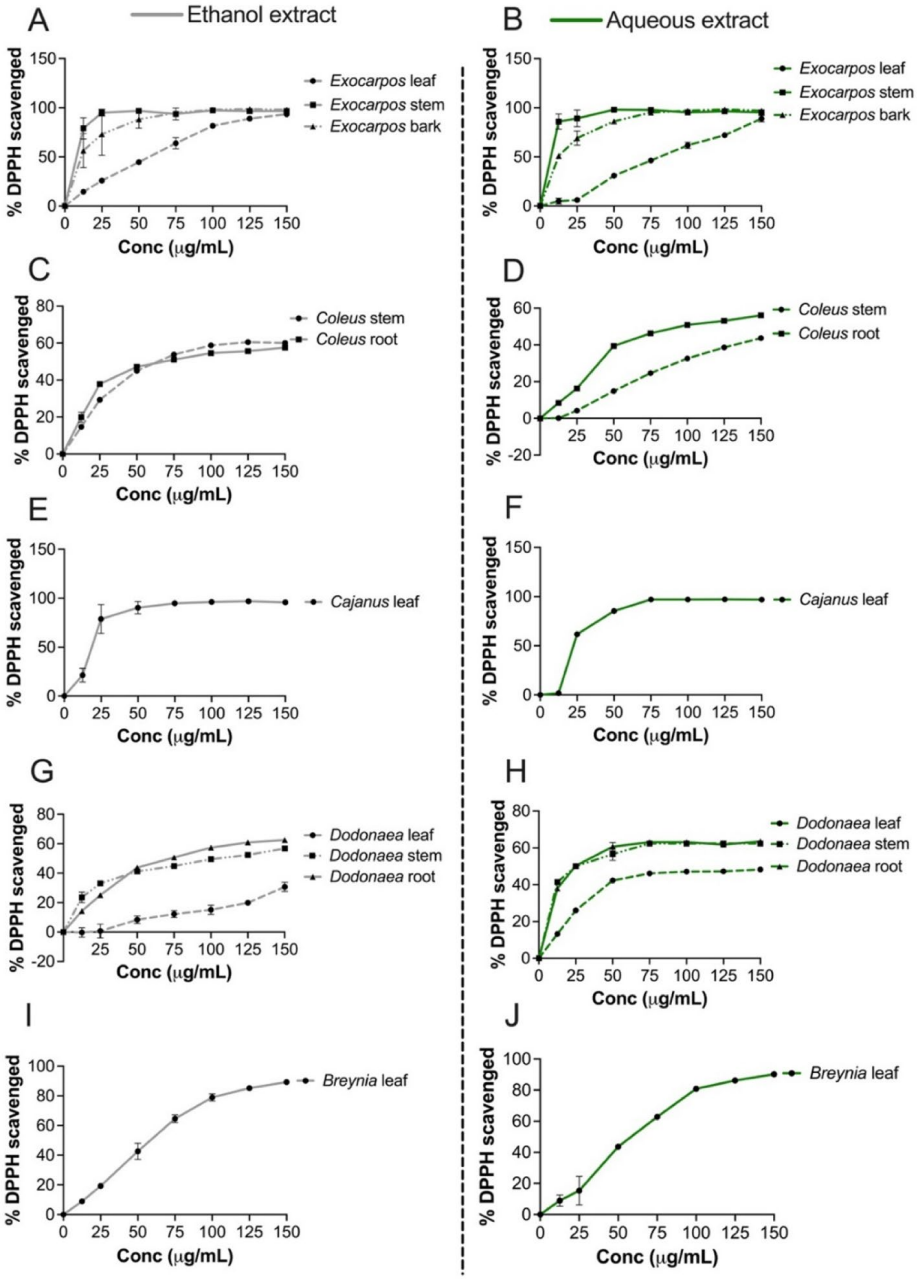
Antioxidant activities of different parts of five medicinal plants extracted using ethanol and water. Results were expressed as mean ± SEM of triplicates (*n* = 3). BL-*Breynia oblongifolia* var. *subsessilifolia* leaf; CL-*Cajanus reticulatus* leaf; DL-*Dodonaea lanceolata var. subsessilifolia* leaf; DS-*Dodonaea lanceolata* var. *subsessilifolia* stem; EL-*Exocarpos latifolius* leaf; EW-*Exocarpus latifolius* wood; EB-*Exocarpos latifolius* bark; CS-*Coleus amoenus* stem; CR-*Coleus amoenus* root

The antioxidant activity of the crude extracts (ethanol and aqueous extracts) obtained from different parts of five plants was investigated by determining the half-maximal effective concentration (EC_50_) values from the dose–response curves of a percentage (%) of DPPH-free radical scavenged by the crude extracts (Fig. 4). Based on the EC_50_ values of the crude water and ethanol extracts of the five medicinal plants, the ethanol crude extracts generally showed better antioxidant activity than the aqueous extracts (Table 3).

**Table 3:** EC_50_ values of antioxidant activities of ethanol and aqueous crude extracts of five medicinal plant species

Plant species	Parts used	Crude extracts	EC_50_ value (μg/mL)
*Breynia oblongifolia*	Leaf	Ethanol extract	53.96
		Aqueous extract	56.46
*Exocarpos latifolius*	Leaf	Ethanol extract	51.99
		Aqueous extract	232.80
	Stem	Ethanol extract	7.905
		Aqueous extract	0.024
	Bark	Ethanol extract	8.84
		Aqueous extract	11.21
*Coleus amoenus*	Stem	Ethanol extract	35.61
		Aqueous extract	67.98
	Root	Ethanol extract	30.10
		Aqueous extract	143.40
*Cajanus reticulatus*	Leaf	Ethanol extract	16.31
		Aqueous extract	20.45
*Dodonaea lanceolata* var.* subsessilifolia*	Leaf	Ethanol extract	17.98
		Aqueous extract	297.4
	Stem	Ethanol extract	17.60
		Aqueous extract	34.79
	Root	Ethanol extract	85.33
		Aqueous extract	172.30
Gallic acid*			0.634

^*^Gallic acid was used as standard antioxidant compound. EC_50_ value was determined based on Fig. 4

When the activities of different plant part extracts were compared, the stem and bark showed the best activities. For example, the water and ethanol extracts of *E. latifolius* stem and bark showed best antioxidant activity with EC_50_ values in the ranges of 0.024–11.21 μg/mL compared to its leaf extract that showed EC_50_ values in the ranges of 51.99–232.80 μg/mL. This is consistent with the moderate to high TPC content of *E. latifolius* extract (46.66 ± 11.96 GAE/g extract, Fig. 3). *B. oblongifolia* leaf (128.36 ± 14.09 GAE/g extract) and *C. amoenus* root and stem (119.67 ± 3.03 and 94.23 ± 10.16 GAE/g) showed the highest TPC and these same extracts showed moderate antioxidant activities (Table 3).

When the leaf extracts of different plant species were compared, the ethanol and water extracts of *C. reticulatus* showed the best DPPH-free radical scavenging activity with EC_50_ values of 16.31 and 20.45 μg/mL, respectively. It also showed good antioxidant activity. Having observed similar antioxidant activities in water and ethanol extracts, we chose water extract of five plants for testing the extracts for their anti-inflammatory activities using PBMC assay. This is also partly because the Mbabaram Aboriginal community use water extracts of these medicinal plants to treat wounds, sores and inflammation.

### Effect of crude extracts on cell viability

The cells were first treated with the crude extracts to determine the cell viability/percentage of live/dead cells resulting from treatment. The crude extract was considered toxic if the percentage of dead cells is ≥ 50% [24]. None of the tested crude extracts affected the cell viability, as the percentage of live cells was more than 99% in all treatment groups (data not shown). Thus, the culture supernatant was further analysed to determine the quantity of pro-inflammatory cytokines/chemokines released by the LPS-stimulated PBMCs after treatment with the aqueous crude extracts of five medicinal plants.

### Anti-inflammatory activity of aqueous crude extracts

**Fig. 5:**
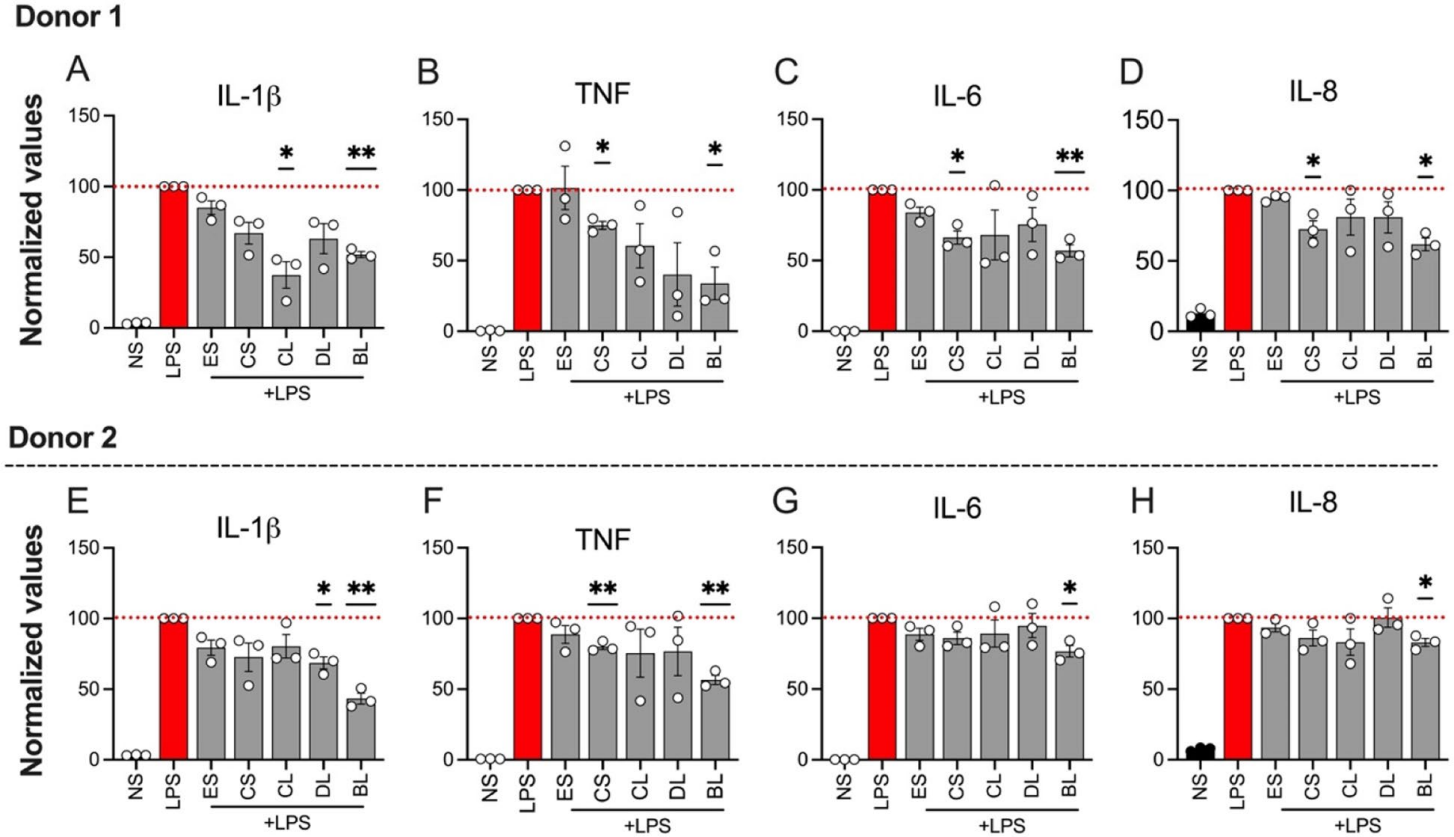
The effects of aqueous crude extracts (100 μg/mL) from five medicinal plants (ES-*Exocarpos latifolius* stem; CS-*Coleus amoenus* stem; CL-*Cajanus reticulatus* leaf; DL-*Dodonaea lanceolata* var. *subsessilifolia* leaf; BL-*Breynia oblongifolia* leaf) on the secretion of pro-inflammatory cytokines by lipopolysaccharide (LPS, 10 ng/mL) stimulated-PBMCs incubated overnight. The levels of secreted cytokines (IL-1β, TNF, IL-6, and IL-8) were determined by flow cytometry and are shown as % of stimulated concentrations of the LPS (100%, red dotted line) for each donor. Bar plots represent the mean normalized cytokine expression of each treatment group (± SEM) performed in triplicates (*n* = 3). Statistical significance was determined by a one-sample *t*-test comparing the LPS-stimulated group to each treatment. ^**^p ≤ 0.0021, ^*^p ≤ 0.0332, and no *p* value = > 0.05

The anti-inflammatory activities of the aqueous crude extracts were measured after treating LPS-stimulated PBMCs with each crude extract at a concentration of 100 μg/mL post overnight incubation. Of the crude extracts tested, *B. oblongifolia* leaf (BL in Fig. 5A-H) showed the best anti-inflammatory activity by significantly reducing the levels of four pro-inflammatory cytokines, namely IL-1β, IL-6, IL-8, and TNF in both PBMC donors (Fig. 5A-H). Interleukin (IL)−1β is considered a key pro-inflammatory cytokine implicated in the inflammatory processes observed in patients with inflammatory bowel disease (IBD), with heightened IL-1β levels often correlating with increased disease severity [5, 10].

*Cajanus reticulatus* leaf (CL) also significantly reduced IL-1β (Fig. 5A), but the suppression was statistically insignificant in the second donor (Fig. 5E). Although IL-1β alone might not be sufficient to incite inflammation, its interaction with other proinflammatory cytokines, such as IL-6 and TNF, has been shown to exacerbate IBD-related inflammation [12]. *C. amoenus* stem extract (CS) also significantly reduced IL-1β, IL-6, and IL-8 in the first PBMC donor (Fig. 5B-D) but significantly suppressed only TNF in the second donor (Fig. 5F). Interleukin (IL)−6 is pleiotropic and plays a multifaceted role in mediating host defence, immunity, disease pathogenesis, and inflammation [6, 15]. Additionally, IL-8, belonging to the CXC family of chemokines, attracts and activates neutrophils at infection sites, initiating the pathogenic cascade seen in IBD [23]. *D. lanceolata var. subsessilifolia* extract also significantly suppressed IL-1β in one of the PBMC donors (Fig. 5E).

## Conclusion

Globally, medicinal plants and traditional medicine remain important in managing and preventing many chronic diseases. Indigenous Australians have vast ethnomedical plant knowledge and have been using it for millennia. In this study, we have evaluated five medicinal plants used by the Mbabaram Aboriginal community in Atherton Tableland in Far-North Queensland for their phytochemical contents, antioxidant and anti-inflammatory properties.

While all five species tested positive for flavonoids, *B. oblongifolia* and *Coleus amoenus* did not contain saponins, and only *Cajanus reticulatus* and *E. latifolius* tested positive for alkaloids. The highest TPC was detected in the ethanol extracts of *B. oblongifolia* leaf and the aqueous extracts of *C. amoenus* root extract. Interestingly, *E. latifolius* stem extract showed the best antioxidant (DPPH-free radical scavenging) activity with EC_50_ value of 0.024 μg/mL, which is even better than gallic acid (0.634 μg/mL) – a standard used for the assay.

Of the five aqueous crude extracts tested, the *B. oblongifolia* leaf extract showed the best suppression of four pro-inflammatory cytokines (IL-1β, IL-6, IL-8, and TNF), which are known for instigating IBD pathogenesis. This result validated the traditional uses of medicinal plants, which is used for treating inflammation-related conditions including wounds and sores. Since this medicinal plant supported strong cell viability and its crude extract consistently showed good suppression of cytokines, we have identified this plant as a candidate for further investigation, including isolation, characterisation and anti-inflammatory testings of pure compounds. It has potential to yield drug lead molecules for developing treatment for inflammation and inflammatory-related diseases such as IBD.

## Data Availability

Data is provided within the manuscript.
